# Environmental Modulation of Physiological Parameters in the Paediatric Intensive Care Unit After Extracorporeal Cardiac Surgery

**DOI:** 10.1111/nicc.70625

**Published:** 2026-08-17

**Authors:** Luna López‐Coleto, Aurora García‐Arcos, Fernando González‐Martín, Esther Hermosilla‐Lloris, Francisco Piedras‐Montilla, Ana M. Durán‐Luengo, Laura Sáez‐García, Ana Díaz‐Vico, Susana Jaraba‐Caballero, Ignacio Morales‐Cané, Francisco José Rodríguez‐Cortés, Pedro Arévalo‐Buitrago, André Sarmento‐Cabral, Raúl Montero‐Yéboles, Pablo J. López‐Soto

**Affiliations:** ^1^ Maimonides Institute for Biomedical Research of Córdoba (IMIBIC), Reina Sofia University Hospital Córdoba Spain; ^2^ Department of Nursing, Pharmacology and Physiotherapy University of Córdoba Córdoba Spain; ^3^ Department of Nursing Reina Sofia University Hospital Córdoba Spain; ^4^ Pediatric Intensive Care Unit Reina Sofia University Hospital Córdoba Spain; ^5^ Department of Cell Biology, Physiology, and Immunology University of Cordoba Córdoba Spain

**Keywords:** critical care nursing, environmental exposure, intensive care unit, paediatric circadian rhythm

## Abstract

**Background:**

The paediatric intensive care unit exposes critically ill children to non‐physiological environmental stimuli, such as noise, artificial light and thermal instability, which may interfere with physiological regulation and circadian organization.

**Aim:**

To examine how environmental conditions in the paediatric intensive care unit (PICU), including noise, lighting and ambient temperature, affect cardiovascular, respiratory and thermoregulatory physiology in paediatric patients admitted after extracorporeal cardiac surgery.

**Study Design:**

This prospective cohort study used continuous environmental monitoring and repeated physiological measurements in a tertiary‐level paediatric intensive care unit. Environmental variables were recorded at 30‐s intervals and aggregated to 15‐min resolution to align with routinely collected physiological data. Associations between environmental exposures and heart rate, blood pressure, respiratory rate, oxygen saturation and rectal temperature were evaluated using generalized additive mixed models (GAMMs) incorporating cyclic terms to account for circadian variation and patient‐level random effects.

**Results:**

Twenty‐seven paediatric patients aged 0–14 years were analysed. Environmental factors showed consistent non‐linear associations with cardiovascular and respiratory parameters. Heart rate and respiratory rate were the most sensitive variables, particularly in relation to visible light exposure, whereas blood pressure, oxygen saturation and rectal temperature showed comparatively attenuated responses. Despite critical illness, several physiological outcomes continued to exhibit significant 24‐h temporal variation throughout the observation period. Age influenced baseline physiology but did not substantially modify the direction of environmental associations. Noise levels consistently exceeded recommended thresholds and lighting patterns lacked a structured day‐night cycle.

**Conclusions:**

PICU environmental conditions were associated with changes in key physiological systems in paediatrics following extracorporeal cardiac surgery and may contribute to altered temporal physiological organization.

**Relevance to Clinical Practice:**

Environmental optimization strategies—particularly structured light–dark cycles and noise reduction warrant further investigation as potential nursing‐led interventions to support physiological regulation and circadian organization in paediatric critical care.

## Impact Statement

- What is known about the topic
  ○ Paediatric intensive care environments expose patients to persistent noise and artificial light.
  ○ Environmental disruption may interfere with circadian organization and physiological regulation in critically ill children.
- What this paper adds
  ○ Noise, light and ambient temperature showed non‐linear associations with cardiovascular and respiratory physiology after paediatric cardiac surgery.
  ○ Heart rate and respiratory rate were the most environmentally sensitive outcomes.
  ○ Associations were consistent across age groups, although younger children showed slightly greater sensitivity to environmental exposures.
  ○ Several physiological outcomes exhibited persistent 24‐h temporal variation despite critical illness, supporting environmental optimization as a potential target for nursing‐led care.

## 1 Introduction

The paediatric intensive care unit (PICU) is a highly demanding environment in which critically ill children are continuously exposed to non‐physiological environmental stimuli, such as elevated noise levels and persistent artificial lighting. These environmental conditions have been associated with poorer physiological adaptation and unfavourable clinical outcomes in paediatric populations, by interfering with essential homeostatic mechanisms [1, 2, 3].

Circadian rhythms coordinate cardiovascular, respiratory and thermoregulatory functions across the 24‐h cycle, generating predictable day‐night variations under physiological conditions [4, 5, 6, 7, 8]. In the PICU, continuous clinical activity and unsynchronized environmental exposures may disrupt this temporal organization, leading to circadian desynchronization, autonomic instability and impaired physiological adaptation [9, 10, 11]. Light, the principal circadian synchronizer (*zeitgeber*), acts through retinal pathways projecting to the suprachiasmatic nucleus to regulate the phase and stability of biological rhythms [12, 13].

Evidence from neonatal intensive care units indicates that lighting patterns directly affect physiological regulation, particularly in preterm newborns. Exposure to continuous or inappropriately intense illumination has been associated with greater haemodynamic instability, characterized by increases in heart rate and blood pressure, as well as reduced oxygenation efficiency, reflecting sustained activation of physiological stress responses [14, 15].

However, there is limited evidence evaluating, in an integrated manner, the impact of the PICU environment on physiological regulation and its temporal organization in critically ill children after extracorporeal cardiac surgery. Cardiac surgery with cardiopulmonary bypass (CPB) temporarily replaces heart and lung function to repair congenital defects but triggers inflammation, endothelial disruption, oxidative stress and neuroendocrine stress responses. In parallel, the PICU environment exposes patients to artificial light, elevated noise, sedation and loss of social time cues. These factors may destabilize central and peripheral circadian oscillators. Melatonin, cortisol and autonomic nervous system activity are key regulators of cardiovascular physiology and exhibit marked circadian rhythmicity. Disruption of these rhythms has been associated with alterations in heart rate regulation, blood pressure, vascular function and cardiovascular outcomes, potentially impairing physiological recovery in critically ill patients [16, 17, 18, 19]. Despite this, integrated chronobiological assessments remain scarce [16, 20]. Few longitudinal studies have captured non‐linear dynamics and residual circadian modulation under real‐world intensive care conditions [16, 17].

## 2 Aim

The aim of the present study was to evaluate the association between environmental conditions in the PICU, including noise, lighting and ambient temperature and cardiovascular, respiratory and thermoregulatory physiological parameters in paediatric patients admitted after cardiac surgery with CPB. A secondary aim was to explore whether these associations differed according to age group (< 2 years vs. ≥ 2 years).

## 3 Design and Methods

### 3.1 Study Design, Participants and Setting

This observational longitudinal study was conducted between January 2024 and August 2025 in a reference PICU of a tertiary‐level hospital in southern Spain. The hospital serves as a regional referral centre for congenital cardiac surgery, and the study was carried out in a recently renovated PICU comprising individual patient rooms designed to optimize environmental conditions and family‐centred care. Patients aged 0–14 years admitted to the PICU with ICD‐10 diagnoses ranging from I00 to I99 [21], corresponding to severe cardiovascular disease, were eligible for inclusion. Only patients who underwent extracorporeal cardiac surgery (e.g., ICD‐10 codes I20.0–I25.4, I25.10, among others), presented no infection at the time of PICU admission, and whose legal guardians signed the informed consent form were included.

The sample size was planned using the GRANMO sample size calculation tool [22], taking into account previous studies conducted by our research group, preliminary data available during the study planning phase, and the annual volume of eligible admissions to the PICU. All eligible patients admitted during the predefined recruitment period were consecutively included.

### 3.2 Ethics Statement

This study was conducted in accordance with the principles of the Declaration of Helsinki and was approved by the Research Ethics Committee of the Province of Córdoba (Reference No. 5502) on 20 December 2023. Written informed consent was obtained from the legal guardians of all participants prior to inclusion in the study. Legal guardians were informed of their right to withdraw from the study at any time without consequences. Whenever the participant's age and clinical condition allowed adequate understanding, the study procedures were explained to the child and their willingness to participate was respected.

### 3.3 Data Collection

Environmental exposures included ambient temperature, noise (dB), visible light intensity, peak light intensity and infrared light. Environmental variables were continuously recorded using a wearable monitoring device (Kronobed, Kronohealth) installed at the head of each patient's bed. Ambient temperature, visible light, peak light intensity, infrared light and noise (dB) were measured using the environmental sensors integrated into the monitoring system. In addition, a temperature probe placed on the patient's back was used for patient temperature monitoring. Environmental variables were sampled every 30 s. Monitoring was performed during two predefined postoperative periods: postoperative day 1 and postoperative day 4.

Physiological outcomes included heart rate, systolic, diastolic and mean arterial blood pressure, respiratory rate, oxygen saturation (SpO_2_) and rectal temperature (when available). These variables were collected approximately every 15 min during routine clinical assessment. Raw data were exported to Excel (xlsx). Environmental data were aggregated into 15‐min intervals to align with physiological measurements.

### 3.4 Data Processing

Data were imported into R (v4.5.2) and processed using *readxl*, *dplyr* and *lubridate*. A conservative quality‐control procedure was applied to exclude technically implausible values based on predefined clinical criteria (see Table S1).

Timestamps were standardized to Central European Time zone and transformed into hours of day (HOD; 0–24 h) to model circadian variation. Visible light variables were log1p‐transformed to stabilize variance. The infrared light variable was excluded due to a highly biased distribution consistent with sensor limitations. The final analytical dataset had a 15‐min temporal resolution.

### 3.5 Data Analysis

Statistical analyses were performed in R (v4.5.2). Environmental exposures and physiological outcomes were merged into a unified analytical dataset with 15‐min temporal resolution.

Associations between environmental exposures and physiological outcomes were evaluated using generalized additive mixed models (GAMMs) implemented in the *mgcv* package. This approach allowed flexible modelling of non‐linear exposure–response relationships in longitudinal repeated measures data.

Separate models were fitted for heart rate, systolic, diastolic and mean arterial blood pressure, respiratory rate, oxygen saturation (SpO_2_) and rectal temperature.

Environmental predictors included ambient temperature, noise (dB) and visible light intensity (log1p‐transformed). Ambient temperature and noise were modelled using penalized cubic regression splines. Hour of day (0–24 h) was modelled using a cyclic cubic spline to account for endogenous circadian modulation independent of environmental exposures.

To evaluate short‐term temporal dynamics, visible light was modelled using distributed lag smooth terms at 15, 30, 45 and 60 min, allowing simultaneous estimation of delayed associations within a one‐hour exposure window.

To account for within‐patient clustering, a patient‐level random intercept was included in all models. Given the serial correlation inherent to 15‐min repeated measurements, models incorporated an autoregressive correlation structure of order 1 (AR (1)) to address residual temporal autocorrelation.

Models were adjusted for prespecified clinical covariates when sufficient variability was present, including age (continuous), PIM2 score, RASS, pacemaker status, vasopressor use and mortality. Primary models evaluated environmental associations independent of PICU length of stay. Sensitivity analyses included PICU day as a linear covariate to assess progressive physiological evolution during PICU admission.

Models were estimated to use restricted maximum likelihood (REML). Model performance was assessed using adjusted *R* ^2^ and deviance explained. Statistical significance was defined as a two‐sided *p* value < 0.05. Missing physiological observations occurred due to routine clinical care variability in monitoring practices. Analyses were conducted using complete‐case data within each model, and no imputation procedures were applied.

To enhance clinical interpretability, predicted differences between the 25th and 75th percentiles (P25–P75) of environmental exposures were calculated from temporally adjusted models. Given the developmental maturation of circadian rhythms and physiological regulation during early childhood, age was considered a relevant factor in the analytical strategy. Additional stratified analyses were conducted by age group (< 2 vs. ≥ 2 years).

Sensitivity analyses were conducted using peak light intensity as an alternative light metric available in the dataset, given that visible light was quantified as bedside illuminance (lux) without spectral weighting or eye‐level calibration. All GAMM specifications—including smooth terms, distributed lags (15–60 min), random intercept, AR (1) structure and clinical covariate adjustment—were retained. Results from these models are presented in Tables S3 and S4.

## 4 Results

### 4.1 Patient Characteristics

A total of 27 paediatric patients were included (mean age 3.56 ± 4.0 years; 74% female). The most common diagnoses were tetralogy of Fallot (22%) and atrioventricular canal defect (19%), followed by ventricular and atrial septal defects (15% each). All patients required mechanical ventilation; 96% were sedated, 70% received temporary pacing and 67% required vasoactive support. Mean predicted mortality (PIM2) was 3.79% (Table 1).

**TABLE 1:** Baseline characteristics of paediatric patients admitted to the PICU.

Age	3.56 (4.0)
Gender	
Male	7 (26%)
Female	20 (74%)
Clinical diagnosis	
Tetralogy of Fallot	6 (22%)
Transposition of great arteries (TGA)	3 (11%)
Ventricular Septal Defect (VSD)	4 (15%)
Atrioventricular canal defect (AV canal)	5 (19%)
Atrial septal defect	4 (15%)
Pulmonary stenosis	1 (4%)
Other congenital heart diseases	4 (15%)
Mechanical ventilation	27 (100%)
PICU Length of stay	16.96 (19.75)
Hospital discharge (survival)	22 (88%)
PIM2	3.79%
RASS score, median [IQR]	−5 [−5, −4]
Patient with pacemaker (%)	19 (70%)
Sedated patient (%)	26 (96%)
Patients on vasopressors or inotropes (%)	18 (67%)

*Note:* Data are presented as mean (standard deviation) or number of patients (%), as appropriate. Age and PICU length of stay are expressed as mean (SD). Each patient was classified according to a single primary cardiac diagnosis corresponding to the main surgical indication. Percentages may not sum to 100% due to rounding.

Abbreviations: AV canal, atrioventricular canal defect; PICU, Paediatric intensive care unit; PIM2, Paediatric Index of Mortality 2; RASS, Richmond Agitation–Sedation Scale; TGA, transposition of the great arteries; VSD, ventricular septal defect.

Laboratory findings from PICU admission to the 4th day reflected the expected postoperative inflammatory and haemodilution profile, with reductions in haemoglobin, haematocrit, leukocyte counts and electrolytes, alongside improvement in hepatic markers, while renal and coagulation parameters remained stable (Table S2).

### 4.2 Descriptive Statistics of Physiological Parameters

Physiological variables are summarized in Table 2. Most parameters did not follow a normal distribution. Heart and respiratory rates exhibited moderate variability throughout the recording period, whereas oxygen saturation remained generally stable, with limited fluctuations. Blood pressure values were within clinically acceptable ranges and showed moderate temporal variability. Rectal temperature, available in fewer observations, showed a narrow interquartile range, indicating limited thermal fluctuations during the PICU length of stay.

**TABLE 2:** Environmental and physiological variables recorded in paediatric patients admitted to the PICU, by time of day.

Variable	Mean ± SD	Median [IQR]	Min–Max	Day median [IQR]	Night median [IQR]
Environmental variables					
Noise (dB)	70.2 ± 9.5	66.8 [63.6–72.6]	57.9–103.7	67.9 [64.4–74.6]	65.8 [63–69.6]
Visible light (lux)	29.6 ± 104.3	4.6 [0.6–12.2]	0–1000	7.7 [3–16.1]	1.8 [0.3–7.2]
Peak light (lux)	35.4 ± 126.1	4.8 [0.6–12.7]	0–1510	8.3 [3.3–17.2]	2 [0.3–7.8]
Ambient temperature (°C)	24.4 ± 1.3	24.4 [23.6–25.1]	20.9–34.8	24.3 [23.6–25]	24.4 [23.7–25.2]
Physiological variables					
Heart rate (bpm)	123 ± 23.2	124 [106–140]	37–196	125 [108–141]	123 [104–139]
Respiratory rate (breaths/min)	27.6 ± 8	27 [22–32]	5–73	27 [22–31]	27 [22–32]
SpO_2_ (%)	95.3 ± 5.9	97 [94–99]	70–100	98 [94–100]	97 [94–99]
Diastolic BP (mmHg)	54.1 ± 9.1	53 [48–59]	34–120	53 [48–59]	53 [48–59]
Mean arterial pressure (mmHg)	67.7 ± 9.5	67 [61–73]	34–156	66 [61–74]	68 [63–73]
Systolic BP (mmHg)	93.2 ± 15.7	92 [82–104]	45–184	92 [81–103]	93 [83–105]
Rectal temperature (°C)	35.5 ± 3.3	36.6 [35.9–37]	25–41	36.6 [35.7–37.1]	36.7 [36.2–36.9]

*Note:* Data are presented as mean ± standard deviation (SD), median [interquartile range, IQR] and range (min–max). Day period was defined as 08:00–19:59 and night period as 20:00–07:59 (local time). Environmental variables were recorded continuously in the PICU room. Physiological variables correspond to routinely monitored vital signs.

Abbreviations: BP: blood pressure; PICU: Paediatric Intensive Care Unit; SpO_2_: peripheral oxygen saturation.

### 4.3 Descriptive Statistics of Environmental Conditions

Environmental variables are summarized in Table 2. Most parameters showed non‐normal, right‐skewed distributions, particularly light‐related measures. Noise levels remained consistently elevated throughout the 24‐h period, with only minimal day‐night differences. Visible light exposure was generally low, though intermittent high‐intensity peaks were recorded, leading to marked variability. Daytime light levels were higher than nocturnal values, but nocturnal exposure was not completely abolished. In contrast, ambient temperature remained stable across the recording period (daytime median 23.3°C vs. nighttime median 24.4°C), indicating minimal circadian variation within the PICU environment.

### 4.4 Generalized Additive Mixed Models

**FIGURE 1:**
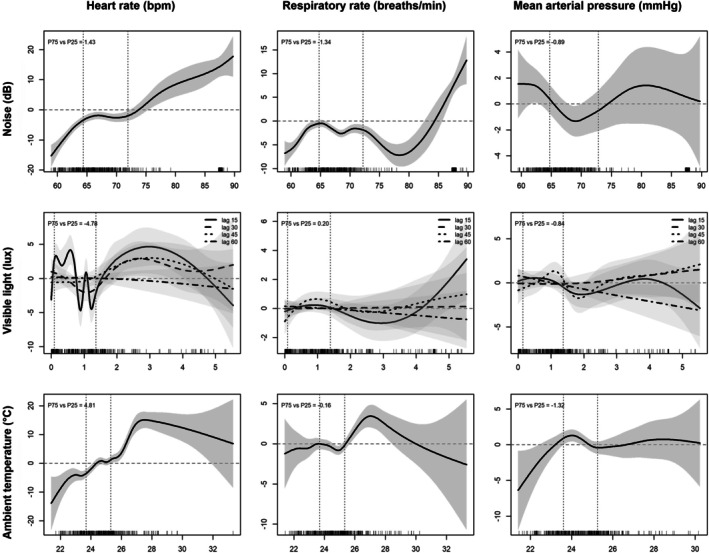
Exposure–response relationships between environmental factors and cardiovascular and respiratory parameters in paediatric intensive care unit (PICU). Generalized additive mixed models (GAMMs) depict adjusted partial effects of noise (dB), visible light (lux) and ambient temperature (°C) on heart rate (bpm), respiratory rate (breaths/min) and mean arterial pressure (mmHg). Shaded bands represent 95% confidence intervals and rug plots illustrate exposure distributions. Vertical dashed lines indicate the 25th and 75th percentiles of each exposure; panel annotations correspond to the estimated difference between these percentiles (ΔP75–P25). Models incorporated a cyclic spline for hour of day and patient‐level random intercepts and were adjusted for age and PICU day. The *x*‐axis represents the environmental exposure variable (noise, visible light or ambient temperature) in its corresponding unit, whereas the *y*‐axis represents the adjusted partial effect estimated by the GAMM on the corresponding physiological outcome. Positive and negative values indicate the direction and magnitude of the modelled association.

GAMMs were used to evaluate non‐linear associations between PICU environmental exposures and physiological outcomes using 15‐min longitudinal data (Figure 1). All models included ambient temperature, visible light intensity (distributed lags 15–60 min), noise, a cyclic smooth term for hour of day, a patient‐level random intercept and AR [1] correlation to account for serial dependence. Sensitivity models additionally included PICU Day. A summary of model fit and significant predictors is presented in Table S3. Detailed age‐adjusted model specifications and percentile‐based contrasts are presented in Tables S5–S7.

#### 4.4.1 Cardiovascular Variables

Following quality control and 15‐min aggregation, data from 2248 heart rate, 2006 systolic blood pressure, 2004 diastolic blood pressure and 2130 mean arterial pressure observations were analysed. The model fitted the data very well for heart rate and was moderate for arterial pressure variables.

Ambient temperature and lagged visible light were significantly associated with all cardiovascular outcomes, whereas noise showed outcome‐specific effects. Heart rate demonstrated the strongest environmental sensitivity, particularly to visible light at 15 min. In contrast, arterial pressure responses were smaller in magnitude and tended to emerge at longer lags (45–60 min).

Significant 24‐h temporal variation was detectable for heart rate but was weaker or absent for arterial pressure variables. When PICU day was included, all cardiovascular outcomes showed significant positive temporal trends, reflecting progressive postoperative haemodynamic evolution.

Modelled percentile contrasts indicated that heart rate exhibited the largest predicted change across environmental exposure ranges, particularly for visible light and ambient temperature. Arterial pressure variables showed comparatively smaller changes, generally within 1–2 mmHg.

#### 4.4.2 Respiratory Variables

After quality control, data from 2118 respiratory rate and 2055 oxygen saturation observations were included. Model performance was good for respiratory rate and excellent for oxygen saturation.

Ambient temperature, visible light and noise showed non‐linear associations with both respiratory outcomes. Respiratory rate displayed a significant association with visible light at 15 min, whereas oxygen saturation exhibited a more distributed lag pattern, with significant effects at 15 and 60 min.

Significant 24‐h temporal variation remained evident in both respiratory variables. Including PICU day showed positive trends over time for respiratory rate and oxygen saturation, indicating ongoing clinical stabilization.

Percentile‐based contrasts showed modest but measurable changes in respiratory rate in response to visible light and noise. Oxygen saturation exhibited smaller amplitude changes across environmental exposure ranges.

#### 4.4.3 Central Body Temperature

A total of 731 rectal temperature observations were analysed. The model fit was excellent across both the primary and temporally adjusted specifications.

Visible light showed a delayed association with rectal temperature, with the strongest effect at the 30‐min lag. Ambient temperature and noise were not independently associated after adjustment. A significant time‐of‐day effect remained evident for rectal temperature, and PICU Day was not significantly associated with this outcome. Predicted temperature changes across environmental exposure ranges were small compared with cardiovascular and respiratory outcomes, consistent with tighter thermoregulatory control.

## 5 Age‐Stratified Analyses

**FIGURE 2:**
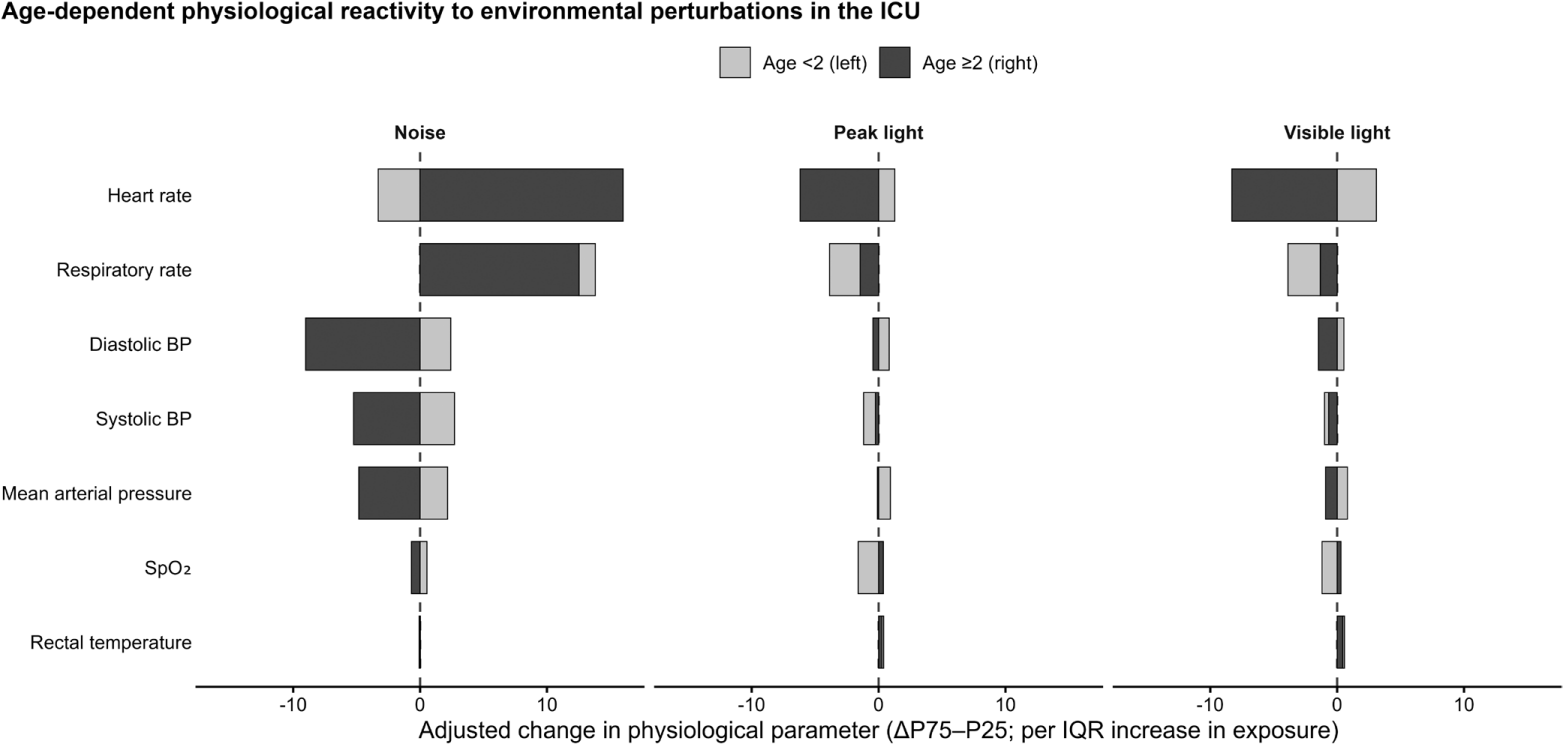
Age‐dependent physiological reactivity to environmental perturbations in the paediatric intensive care unit. Tornado plot showing the adjusted change (Δ) in physiological parameters associated with an interquartile range (IQR), increase in environmental exposures (noise, peak light and visible light), stratified by age group (< 2 years and ≥ 2 years). Bars represent model‐estimated differences between the 75th and 25th percentile of exposure (ΔP75–P25). For visualization purposes, estimates for children < 2 years are displayed on the left side and those for children ≥ 2 years on the right side. Physiological parameters are expressed in their conventional clinical units: heart rate (beats per minute), respiratory rate (breaths per minute), blood pressure (mmHg), oxygen saturation (%) and rectal temperature (°C).

Age‐stratified analyses (< 2 vs. ≥ 2 years) showed broadly consistent environmental exposure–response patterns across age groups, supporting the robustness of the primary findings (Figure 2; Tables S8A–S9B). Ambient temperature, visible light and noise were significantly associated with cardiovascular and respiratory variables in both strata.

Twenty‐four‐hour temporal variation was more consistently observed in children < 2 years across cardiovascular outcomes, whereas in older children it was not detectable for all variables. When PICU day was included as a linear covariate, age‐specific temporal trends in heart rate emerged, with an increase in older children and a decrease in younger patients; however, environmental associations remained stable in both groups.

Percentile‐based contrasts (ΔP75‐P25) confirmed that heart rate and respiratory rate were the most environmentally sensitive outcomes in both age groups (Figure 2; Table S10A,B). Visible light was associated with reductions in heart rate and increases in respiratory rate in both strata, while noise and ambient temperature produced the largest cardiovascular changes. In contrast, blood pressure, oxygen saturation and rectal temperature exhibited comparatively small variations, consistent with tighter physiological buffering.

Overall, although effect magnitudes were slightly greater in younger children for selected outcomes, age did not substantially modify the direction of environmental associations.

## 6 Discussion

Based on the applied methodological approach, associations were identified between environmental conditions in the PICU and cardiovascular, respiratory and thermal physiological parameters in children following extracorporeal cardiac surgery. Heart and respiratory rate were the most sensitive variables—particularly in relation to light exposure, with associations concentrated at short time lags (approximately 15 min)—whereas blood pressure, oxygen saturation and body temperature exhibited more attenuated and temporally heterogeneous responses, often emerging at longer lags (30–60 min), suggesting greater physiological buffering. Despite critical illness, significant 24‐h temporal variation remained evident across most outcomes, although its magnitude varied according to outcome type and model specification. Although age influenced baseline physiology and clinical context, it did not substantially modify the direction of environmental associations, underscoring the PICU environment as a potentially modifiable determinant of paediatric physiological regulation.

The differential sensitivity across physiological systems likely reflects their underlying regulatory mechanisms. Fast‐response parameters such as heart and respiratory rate are predominantly modulated by the autonomic nervous system and therefore respond rapidly to external stimuli [23, 24]. Exposure to noise has been associated with acute autonomic activation, manifested as increases in heart rate and respiratory irregularities [25, 26]. Similarly, elevated light exposure—both during the day and at night—has been linked to changes in heart rate dynamics without substantial alterations in more tightly regulated parameters [27]. The short‐latency associations observed in our distributed‐lag models further support a rapid autonomic mediation of light‐related responses. In contrast, arterial pressure, oxygen saturation and thermoregulation depend on integrated control systems—including baroreceptor reflexes, vascular tone, ventilation–perfusion balance and hypothalamic regulation—which buffer acute environmental perturbations [28, 29, 30, 31, 32, 33]. The more delayed and variable light‐lag patterns observed for these outcomes are consistent with slower integrative regulatory pathways.

Noise levels in our PICU consistently exceeded international recommendations. The World Health Organization advises hospital noise levels below 35–40 dB, and the American Academy of Paediatrics recommends ≤ 45 dB during both day and night [34, 35, 36, 37]. However, environmental monitoring revealed persistently elevated noise exposure with minimal circadian variation. Likewise, lighting patterns were characterized by low baseline illumination and intermittent high‐intensity peaks, suggesting the absence of a stable and structured light–dark cycle. Such irregular exposure patterns may contribute to short‐term physiological fluctuations and impaired temporal organization.

These findings highlight opportunities for environmental optimization in the PICU. Reducing unnecessary noise, minimizing nighttime disturbances and promoting structured light–dark cycles may help preserve temporal environmental cues. Although further evaluation is required, these interventions represent feasible targets for nursing‐led environmental management.

Taken together, these findings characterize the PICU as a potentially chronodisruptive environment. Chronodisruption has been associated with adverse cognitive, metabolic, cardiovascular and immune outcomes in paediatric populations [38, 39, 40, 41, 42]. Importantly, the persistence of 24‐h temporal variation in several physiological outcomes suggests that daily physiological organization may not be completely abolished during critical illness. However, because direct circadian biomarkers such as melatonin, cortisol, sleep–wake cycles or molecular clock markers were not assessed, these findings should not be interpreted as definitive evidence of preserved circadian rhythmicity. Our results suggest that vulnerability may be particularly relevant in younger children, whose circadian organization remains immature and highly dependent on environmental cues, particularly light [43, 44]. During early life, the consolidation of stable 24‐h rhythms is still developing, making external temporal signals critical for physiological synchronization [42, 43].

A previous study has demonstrated that structured cyclic lighting promotes circadian maturation, improved functional organization and better weight gain, whereas constant illumination has been associated with physiological stress and disorganization [44]. Similarly, randomized interventions aimed at reducing environmental noise have shown improvements in weight gain and neurodevelopmental outcomes [45], as well as acute reductions in heart and respiratory rate and improved sleep organization [46]. Cyclic lighting interventions have likewise been associated with clearer day‐night differentiation and improved physiological stability [47]. Our findings extend this evidence by demonstrating temporally structured, lag‐specific physiological responses in children following cardiac surgery under real‐world intensive care conditions. In addition to environmental strategies, chronobiological interventions such as exogenous melatonin may warrant further evaluation as potential approaches to support circadian organization and physiological recovery in paediatric critical care settings.

## 7 Limitations

This study has several limitations. First, the relatively small sample size (*n* = 27) may limit statistical power and the precision of effect estimates, although the high number of repeated measures partially mitigates this constraint. Second, the single‐centre design may reduce external validity, as environmental conditions and clinical practices can differ across PICUs. Third, although environmental monitoring was performed using a standardized wearable device, potential sensor‐related limitations—such as measurement bias in specific light channels and restricted spatial sampling around the patient's bed—may have influenced exposure quantification. Light exposure was quantified as bedside illuminance and does not necessarily reflect melanopic‐equivalent or eye‐level photic dose, particularly in sedated children with closed eyelids. However, sensitivity analyses using peak light intensity yielded results comparable to those using visible light intensity, partially mitigating this limitation. Finally, as an observational study, causal inference cannot be established despite statistical adjustment for relevant clinical covariates. Future multicentre and interventional studies are needed to confirm these findings and to determine whether structured environmental modifications can improve physiological and circadian stability in paediatric critical care.

## 8 Implications for Practice and Further Research

Environmental management in the PICU should be considered a potentially modifiable component of postoperative care. Strategies aimed at reducing noise and promoting a structured light–dark cycle may help support physiological regulation and the maintenance of appropriate day‐night environmental cues.

Future multicentre interventional studies should assess whether environmental optimization, alone or combined with chronobiological interventions such as melatonin administration, leads to measurable improvements in circadian regulation, physiological recovery and clinical outcomes in this population.

## 9 Conclusions

This study demonstrates that paediatric patients undergoing cardiac surgery and admitted to the PICU are continuously exposed to an adverse environmental context, characterized by persistently elevated noise levels, suboptimal lighting conditions and the absence of a clearly structured day‐night cycle. Using generalized additive models, these environmental factors were significantly associated with changes in key physiological parameters, including heart rate, respiratory rate, blood pressure and core body temperature.

Although 24‐h temporal variation in these variables was modest, a substantial proportion of their daily variability was explained by PICU environmental patterns, indicating a relevant degree of circadian disruption in this particularly vulnerable population.

Collectively, these findings support further investigation of environmental optimization strategies, such as reducing noise and structuring light exposure, to better understand their potential role in supporting physiological regulation and circadian organization in paediatric critical care patients.

## Funding

The authors have nothing to report.

## Ethics Statement

This study was conducted in accordance with the Declaration of Helsinki and was approved by the Research Ethics Committee of the Province of Córdoba (Ref. 5502). Written informed consent was obtained from the legal guardians of all participants prior to inclusion in the study. Legal guardians were informed of their right to withdraw from the study at any time without consequences.

## Conflicts of Interest

The authors declare no conflicts of interest.

## Supporting information


**Table S1:** Quality‐control thresholds applied prior to statistical analysis.
**Table S2:** Comparison of laboratory parameters between day 1 and day 4.
**Table S3:** Summary of GAMMs evaluating associations between PICU environmental conditions and physiological outcomes.
**Table S4:** Summary of peak‐light GAMMs with PICU Day adjustment.
**Table S5:** Percentile‐based contrasts (ΔP75–P25) from age‐adjusted GAMMs comparing bedside visible light and peak light.
**Table S6:** Summary of age‐adjusted generalized additive models (GAMMs) including distributed lags of visible light and PICU Day.
**Table S7:** Summary of age‐adjusted generalized additive mixed models (GAMMs) including distributed lags of peak light exposure and PICU Day.
**Table S8:** Percentile‐based contrasts (ΔP75–P25) from age‐adjusted GAMMs comparing visible light and peak light exposure.
**Table S9A:** Age‐stratified generalized additive mixed models (GAMMs) in children aged ≥ 2 years.
**Table S9B:** Age‐stratified generalized additive mixed models (GAMMs) in children aged < 2 years.
**Table S10A:** Percentile‐based contrasts (ΔP75–P25) from age‐stratified (≥ 2 years) age‐adjusted GAMMs comparing visible light and peak light exposure.
**Table S10B:** Percentile‐based contrasts (ΔP75–P25) from age‐stratified (< 2 years) age‐adjusted GAMMs comparing visible light and peak light exposure.

## Data Availability

The data that support the findings of this study are available from the corresponding author upon reasonable request.
