# Supplementary material for: Environmental Modulation of Physiological Parameters in the Paediatric Intensive Care Unit After Extracorporeal Cardiac Surgery
Source: Nurs Crit Care. 2026 Aug 17;31(5):e70625. doi: 10.1111/nicc.70625 (PMC13479336; doi:10.1111/nicc.70625)
Supplement: Supplementary file 1 [file NICC-31-0-s001.docx]

**Supplementary Material.**

**S1. Data Processing and Quality-Control Criteria.**

Prior to descriptive analyses and model fitting, a predefined quality-control (QC) procedure was applied to exclude technically implausible values and potential sensor artifacts.

Environmental variables were screened to remove saturation codes and extreme out-of-range values consistent with device limitations. Physiological variables were filtered according to clinically plausible ranges. All exclusions were applied prior to 15-minute aggregation and statistical modeling.

**Table S1. Quality-control thresholds applied prior to statistical analysis.**

| **Variable** | **Accepted analytical range** |
| --- | --- |
| **Environmental variables** |  |
| Visible light (lux) | 0-1000 |
| Peak light (lux) | 0-2000 |
| Infrared light | ≥0 (sensor artifacts excluded) |
| Ambient temperature (°C) | 15-35 |
| Noise (dB) | 30-120 |
| **Physiological variables** |  |
| Heart rate (bpm) | 30-220 |
| Respiratory rate (breaths/min) | 4-80 |
| Oxygen saturation (%) | 70-100 |
| Systolic blood pressure (mmHg) | 40-250 |
| Diastolic blood pressure (mmHg) | 20-130 |
| Mean arterial pressure (mmHg) | 30-180 |
| Rectal temperature (°C) | 25-43 |

***Table S1.* *Quality-control thresholds applied prior to statistical analysis.*** *Thresholds were predefined according to device specifications and clinically plausible physiological ranges. Values outside these limits were set to missing (NA) prior to 15-minute aggregation and model fitting. Sensor saturation codes (≥32000 or 65535) were removed before range filtering.*

**S2. Comparison of laboratory parameters between Day 1 and Day 4.**

|  |  | ***Day 1*** | ***Day 4*** |
| --- | --- | --- | --- |
| Hemoglobin | g/dL | 11.98 ± 2.15 | 10.43 ± 1.50 (*) |
| Hematocrit | % | 36.05 ± 6.41 | 31.21 ± 4.73(*) |
| Leukocytes | x10^3/µL | 13.05 ± 5.43 | 8.85 ± 4.06(*) |
| Neutrophils | x10^3/µL | 8.87 ± 3.46 | 5.23 ± 2.42(**) |
| Glucose | mg/dL | 111.50 [91.25–138.25] | 105.73 ± 31.96 |
| Creatinine | mg/dL | 0.44 ± 0.17 | 0.45 ± 0.16 |
| Sodium | mEq/L | 143.81 ± 4.55 | 139.87 ± 4.17(**) |
| Potassium | mEq/L | 4.33 ± 0.60 | 3.95 ± 0.50 |
| Chloride | mg/dL | 109.96 ± 3.50 | 104.87 ± 4.34(**) |
| Calcium | mg/dL | 8.93 ± 0.58 | 8.72 ± 0.52 |
| Total bilirubin | mg/dL | 0.70 [0.60–1.12] | 0.39 ± 0.18(**) |
| AST (GOT) | U/L | 121.50 [59.75–249.00] | 65.00 [32.00–123.00](*) |
| ALT (GPT) | U/L | 19.00 [14.25–28.75] | 15.00 [10.50–27.25] |
| Prothrombin time | % | 65.91 ± 9.73 | 71.67 ± 18.04 |
| INR |  | 1.34 [1.24–1.43] | 1.24 [1.11–1.31] |
| Fibrinogen |  | 316.13 ± 76.24 | 349.67 ± 115.73 |
| C-reactive protein | mg/L | 60.36 ± 34.38 | 57.50 [48.75–85.55] |

***S2. Comparison of laboratory parameters between Day 1 and Day 4.*** *Data are presented as mean ± standard deviation or median [interquartile range], according to data distribution. Normality was assessed using the Shapiro–Wilk test. Between-group comparisons were performed using the unpaired Welch’s t-test for normally distributed variables and the Mann–Whitney U test for non-normally distributed variables* p < 0.05, ** p < 0.01*

**S3. Extended Statistical Methods.**

All supplementary analyses follow the primary modeling strategy described in the main manuscript. Briefly, associations between paediatric intensive care unit (PICU) environmental exposures and routinely monitored physiological outcomes were evaluated using generalized additive mixed models (GAMMs) to capture potentially non-linear exposure–response relationships in 15-minute longitudinal data while accounting for repeated measures and serial autocorrelation.

***Model Structure.***

For each outcome (heart rate, systolic, diastolic, and mean arterial blood pressure, respiratory rate, oxygen saturation, and rectal temperature), the model included smooth terms for ambient temperature and noise (dB), specified as contemporaneous exposures using penalized cubic regression splines.

Visible light intensity was log1p-transformed to reduce skewness and stabilize variance. To evaluate short-term temporal dynamics, visible light was modeled using a distributed lag specification including lagged exposures at 15, 30, 45, and 60 minutes. This approach allowed simultaneous estimation of delayed light–response associations within a one-hour exposure window.

Residual circadian modulation was represented using a cyclic cubic regression spline for hour of day (0–24 h). Repeated measures were handled through a patient-level random intercept (random-effect smooth for patient ID).

Lagged variables were generated within each patient after chronological ordering of observations. Lag values were retained only when the preceding time intervals were consistent with the expected 15-minute resolution (tolerance ±2 minutes). When temporal gaps were detected, lagged values were set to missing to avoid propagation of exposure misalignment.

***Autocorrelation Structure.***

Given the serial dependence inherent to 15-minute repeated physiological measurements, models incorporated an autoregressive correlation structure of order 1 (AR(1)). Models were fitted using the bam() function (mgcv package, R) with fast restricted maximum likelihood (fREML) estimation. A fixed autoregressive parameter (ρ = 0.3) was specified, and new autoregressive segments were initiated at the start of each patient series and after identified time gaps.

***Estimation and inference.***

Models were estimated using fREML within the mgcv framework in R (v4.5.2). Statistical significance of smooth terms was assessed using approximate tests provided by mgcv. Model performance was summarized using adjusted R² and deviance explained. Diagnostic procedures, including basis adequacy and residual inspection, were performed for all fitted models.

To facilitate clinical interpretation, percentile-based contrasts were calculated as the expected change in the outcome between the 25th and 75th percentiles (P25–P75) of each environmental exposure, holding other covariates at typical values. Predictions were obtained at the population level by excluding the patient-level random-effect term. For distributed-lag light models, both single-lag contrasts and joint contrasts (simultaneous shift of all light lags from P25 to P75) were computed.

***Sensitivity analysis for PICU time.***

Because postoperative physiology evolves during PICU stay, sensitivity models additionally included PICU Day as a linear covariate when the variable was non-constant within a participant. These temporally adjusted models were used to summarize the direction and magnitude of outcome trajectories over time and to verify the robustness of environmental associations.

Additional sensitivity analyses were conducted using peak light intensity (log1p-transformed) as an alternative light metric. All model components—including distributed light lags (15–60 minutes), cyclic circadian term, random intercept, and AR(1) structure—were retained to ensure comparability across specifications.

***S4. Supplementary Results. Peak light sensitivity***

***Cardiovascular variables.***

In sensitivity analyses replacing bedside visible light with peak light intensity (log1p-transformed) and maintaining the distributed-lag structure (15–60 minutes), cardiovascular models showed patterns broadly consistent with the primary specification. Heart rate remained the most environmentally sensitive cardiovascular outcome, showing significant non-linear associations with ambient temperature and noise, and significant peak-light associations concentrated at short lags (15–30 minutes) (Table S3). In contrast, arterial pressure outcomes showed weaker and less consistent peak-light associations; when present, peak-light effects tended to emerge at longer lags (notably a borderline association at 60 minutes for diastolic blood pressure), while temperature effects were more consistently detected across blood pressure outcomes.

After temporal adjustment, PICU Day was positively associated with all cardiovascular outcomes (heart rate and blood pressure), indicating progressive postoperative hemodynamic evolution over the PICU stay, while environmental associations remained generally stable. Vasopressor use was associated with lower arterial pressures in temporally adjusted models, and age was positively associated with diastolic blood pressure.

Across cardiovascular outcomes, the largest modeled contrast was observed for heart rate, which decreased with higher light exposure (Visible light: −6.37 bpm; Peak light: −4.60 bpm) and showed a smaller positive contrast for noise (+1.43 bpm) and a positive contrast for temperature (+4.81 bpm) (Table S4). For arterial pressure outcomes, contrasts were modest: temperature was associated with lower SBP/DBP/MAP (from −1.32 to −2.11 mmHg), noise was associated with small decreases (from −0.89 to −2.31 mmHg), and light effects were near-zero to small (SBP slightly positive; DBP and MAP slightly negative), with broadly similar magnitudes for visible light and peak light (Table S4).

***Respiratory variables.***

In sensitivity analyses replacing bedside visible light with peak light intensity (log1p-transformed) while preserving the distributed-lag structure (15–60 minutes), respiratory outcomes showed robust environmental associations that were broadly consistent with the primary specification. Respiratory rate displayed significant non-linear associations with ambient temperature and noise, and peak-light effects were lag-dependent, with significant associations at short (15 minutes) and intermediate (45 minutes) delays (Table S3). After temporal adjustment, PICU Day showed a significant positive trend in respiratory rate, indicating progressive postoperative respiratory evolution over PICU stay, while the environmental smooth associations and the residual circadian component remained detectable.

For oxygen saturation, models showed excellent fit and strong patient-level heterogeneity. Ambient temperature remained significantly associated with SpO₂. Noise showed evidence of association, reaching borderline significance in temporally adjusted models and becoming statistically significant in the primary specification. Peak-light associations were most evident at 15 minutes and 60 minutes (with additional borderline contribution at 30 minutes in temporally adjusted models), indicating a more distributed temporal pattern of light-related modulation compared with respiratory rate. The cyclic circadian term remained significant in both the primary and temporally adjusted models, and PICU Day was positively associated with SpO₂ over time. In temporally adjusted models, death during admission was associated with lower SpO₂ values.

Respiratory rate increased with higher light exposure (Visible light: +1.11 breaths/min; Peak light: +1.07 breaths/min) and decreased with higher noise (−1.34 breaths/min), while the temperature contrast was close to null (−0.16 breaths/min). Oxygen saturation increased with higher light (Visible light: +0.79%; Peak light: +0.86%) but showed minimal temperature effect (+0.08%) and a small negative noise contrast (−0.73%) (Table S4).

***Central body temperature.***

In sensitivity analyses replacing bedside visible light with peak light intensity (log1p-transformed) while preserving the distributed-lag structure (15–60 minutes), rectal temperature models demonstrated excellent overall fit and substantial patient-level heterogeneity. In contrast to respiratory outcomes, ambient temperature and noise were not significantly associated with temperature in either the primary or temporally adjusted specification (Table S3).

Peak-light effects were clearly lag-dependent, with a robust non-linear association observed at 30 minutes in both models, and a borderline contribution at 60 minutes after temporal adjustment. No significant associations were detected at shorter lags (15 minutes) or at 45 minutes (Table S3).

The cyclic circadian term remained statistically significant in both the primary and temporally adjusted models, indicating persistent residual diurnal modulation independent of measured environmental exposures. PICU Day was not significantly associated with temperature, and inclusion of temporal adjustment did not materially alter the magnitude or pattern of peak-light associations.

**Table S3. Summary of GAMMs evaluating associations between PICU environmental conditions and physiological outcomes**

| **Outcome** | **Model fit (R²)** | **Temperature** | **Visible light** | **Noise** | **Light lag pattern** | | **Circadian pattern (Primary/ +PICU Day)** | **PICU Day trend** |
| --- | --- | --- | --- | --- | --- | --- | --- | --- |
| **Heart rate (bpm)** | 0.78 | ✓ | ✓ | ✓ | 15 min (primary) | Yes/Yes | | +3.03 bpm/day |
| **Systolic BP (mmHg)** | 0.56 | ✓ | ✓ | Borderline | 60 min | Yes/No | | +1.77 mmHg/day |
| **Diastolic BP (mmHg)** | 0.29 | ✓ | ✓ | ✓ | 15 and 60 min (weak) | No/No | | +1.68 mmHg/day |
| **Mean arterial pressure (mmHg)** | 0.33 | ✓* | ✓ | ✗ | 45-60 min | No / No | | +1.08 mmHg/day |
| **Respiratory rate (breaths/min)** | 0.60 | ✓ | ✓ | ✓ | 15 min | Yes/Yes | | +0.69 breaths/min/day |
| **Oxygen saturation (%)** | 0.84 | ✓ | ✓ | ✓* | 15 and 60 min | Yes/Yes | | +0.44%/day |
| **Rectal temperature (°C)** | 0.91 | ✗ | ✓ | ✗ | 30 min | Yes/Yes | | NS |

***Table S3. Summary of GAMMs evaluating associations between PICU environmental conditions and physiological outcomes.*** *Model fit is expressed as the adjusted R², which represents the proportion of outcome variability explained by the generalized additive models (GAMs). All models included ambient temperature, visible light intensity modeled using distributed lags (15–60 minutes), noise levels, a cyclic smooth term for hour of day (circadian component), a patient-level random intercept to account for repeated measures, and an AR(1) structure to account for residual autocorrelation.✓ indicates a statistically significant association (p < 0.05).*

*✓* indicates a statistically significant but weaker or less consistent association across model specifications. ✗ indicates no statistically significant association (p ≥ 0.05). Borderline indicates marginal statistical significance (0.05 ≤ p < 0.10).“Light lag pattern” refers to the lag(s) within the 15–60-minute window showing the strongest or most consistent association in distributed-lag models. “Circadian pattern (Primary / +PICU Day)” refers to the presence of a significant 24-hour cyclic component in the model without PICU Day (Primary) and after inclusion of PICU Day as a linear covariate (+PICU Day). “Yes” indicates statistically significant circadian modulation; “No” indicates absence of significant rhythmicity.“PICU Day trend” represents the estimated linear change in the outcome per additional day of ICU stay in temporally adjusted models. NS indicates a non-significant temporal trend.*

***Table S4. Summary of peak-light GAMMs with PICU Day adjustment.***

| **Outcome** | **n** | **Adjusted R²** | **Deviance explained** | **Temperature** | **Noise** | **Peak light (distributed lags 15–60 min)** | **Circadian term (HOD)** | **PICU Day (β/day)** |
| --- | --- | --- | --- | --- | --- | --- | --- | --- |
| Heart rate (bpm) | 2248 | 0.774 | 77.8% | ✓ | ✓ | ✓ at 15 & 30 min | Yes | +3.02 bpm/day |
| Systolic BP (mmHg) | 2006 | 0.562 | 56.9% | ✓ | Borderline | NS | NS | +1.76 mmHg/day |
| Diastolic BP, (mmHg) | 2004 | 0.291 | 30.2% | ✓ | NS | Borderline at 60 min | NS | +1.68 mmHg/day |
| Mean arterial pressure (mmHg) | 2130 | 0.324 | 33.5% | ✓ | NS | NS | NS | +1.06 mmHg/day |
| Respiratory rate (breaths/min) | 2118 | 0.598 | 60.6% | ✓ | ✓ | ✓ at 15 and 45 min | Yes | +0.67 breaths/min/day |
| Oxygen saturation (%) | 2055 | 0.841 | 84.5% | ✓ | Borderline | ✓ at 15 and 60 min | Yes | +0.46 %/ day |
| Rectal temperature (ºC) | 731 | 0.910 | 91.4% | NS | NS | ✓ | Yes | NS |

***Table S4. Summary of peak-light GAMMs with PICU Day adjustment.*** *AR(1)-adjusted GAMMs (ρ = 0.3; fREML) including smooth terms for ambient temperature and noise, distributed-lag smooths for peak light (log1p) at 15–60 minutes, cyclic spline for hour of day, patient random intercept, and adjustment for clinical covariates (PIM2, RASS, pacemaker, mortality, vasopressors, age, PICU Day). ✓ indicates p < 0.05; Borderline, 0.05 ≤ p < 0.10; NS, non-significant. PICU Day is expressed as linear change per day.*

**Table S5. Percentile-based contrasts (ΔP75–P25) from age-adjusted GAMMs comparing bedside visible light and peak light.**

| **Outcome** | **Temperature Δ** | **Visible light Δ joint 15–60)** | **Peak light Δ joint 15–60)** | **Noise Δ** |
| --- | --- | --- | --- | --- |
| Heart rate (bpm) | +4.81 | −6.37 | −4.60 | +1.43 |
| Systolic BP (mmHg) | −1.67 | +0.41 | +0.28 | −2.31 |
| Diastolic BP (mmHg) | −2.11 | −0.56 | −0.57 | −1.54 |
| Mean arterial pressure (mmHg) | −1.32 | −0.91 | −1.02 | −0.89 |
| Respiratory rate (breaths/min) | −0.16 | +1.11 | +1.07 | −1.34 |
| Oxygen saturation (%) | +0.08 | +0.79 | +0.86 | −0.73 |
| Rectal temperature (°C) | −0.03 | −0.40 | −0.46 | +0.61 |

***Table S5.*** ***Percentile-based contrasts (ΔP75–P25) from age-adjusted GAMMs comparing bedside visible light and peak light.*** *ΔP75–P25 values represent the model-predicted change in each outcome when the exposure increases from the 25th to the 75th percentile. Temperature and noise contrasts were computed for their contemporaneous terms. Light contrasts correspond to joint distributed-lag shifts (15–60 min), i.e., all four lagged light terms (15, 30, 45, and 60 min) were shifted simultaneously from P25 to P75. Models were age-adjusted and included PICU Day (linear term when non-constant), a cyclic spline for hour of day (HOD), a patient-level random intercept, and AR(1) correction (ρ = 0.3). Estimates are expressed in the natural units of each physiological outcome*

**S5. Age-Adjusted Generalized Additive Models.**

Age was included as a continuous covariate (years) in whole-cohort models to evaluate potential confounding of environmental associations. All models additionally included PICU Day as a linear term when non-constant and a cyclic spline for hour of day (HOD). Detailed model summaries are presented in Table S5 (visible light models) and Table S6 (peak light, sensitivity analysis). Percentile-based contrasts are summarized in Table S7.

***Cardiovascular Variables.***

After quality control and 15-minute aggregation, 2,248 heart rate, 2,006 systolic blood pressure, 2,004 diastolic blood pressure, and 2,130 mean arterial pressure observations were analyzed. Model performance ranged from excellent for heart rate (adjusted R² = 0.781) to moderate for systolic blood pressure (0.563), mean arterial pressure (0.329), and diastolic blood pressure (0.292) (Table S5).

Across cardiovascular outcomes, ambient temperature demonstrated consistent non-linear associations. Noise showed significant effects for heart rate, while arterial pressure variables were less consistently influenced by acoustic exposure. Distributed lag models of visible light revealed temporally structured associations. Heart rate showed significant modulation at 15 minutes, with borderline effects at 30 and 60 minutes. Systolic and diastolic blood pressure exhibited delayed associations, primarily at 60 minutes, whereas mean arterial pressure showed significant effects at both 15 and 60 minutes.

In age-adjusted models including PICU Day, the cyclic circadian component remained significant for heart rate and diastolic blood pressure, whereas systolic and mean arterial pressure did not show robust residual rhythmicity. All cardiovascular variables demonstrated significant positive temporal trends over PICU stay, with increases of +3.02 bpm/day for heart rate, +1.76 mmHg/day for systolic blood pressure, +1.71 mmHg/day for diastolic blood pressure, and +1.12 mmHg/day for mean arterial pressure.

Sensitivity analyses using peak light exposure (Table S6) yielded comparable overall model performance and temporal trends, with heart rate again showing early light responsiveness (15–30 minutes), while arterial pressure responses were attenuated or limited to borderline delayed effects.

Heart rate showed the largest environmental responsiveness, with ΔP75–P25 contrasts of −6.37 bpm (visible light) and −5.14 bpm (peak light), indicating consistent light-related modulation across metrics. Temperature was positively associated with heart rate (+4.81 bpm), while noise showed a modest positive effect (+1.43 bpm). In contrast, arterial pressure variables exhibited small modeled changes (<2 mmHg), with minimal differences between visible and peak light metrics. Overall, light effects were most pronounced for heart rate, whereas blood pressure responses were comparatively limited (Table S7).

***Respiratory Variables.***

After quality control and 15-minute aggregation, 2,118 respiratory rate and 2,055 oxygen saturation observations were analyzed. Model performance was moderate for respiratory rate (adjusted R² = 0.599) and excellent for oxygen saturation (0.841) (Table S5).

Ambient temperature and noise demonstrated significant non-linear associations for both respiratory variables. Distributed lag light models indicated early effects of visible light on respiratory rate (15 minutes) with borderline effects at 45 minutes. Oxygen saturation showed significant associations at both short (15 minutes) and delayed (60 minutes) lags.

The cyclic circadian component remained significant for both respiratory rate and oxygen saturation after age and temporal adjustment. Both variables exhibited significant positive temporal trends during PICU stay (+0.67 breaths/min/day for respiratory rate and +0.45%/day for oxygen saturation).

Sensitivity analyses using peak light exposure (Table S6) demonstrated a similar pattern, although oxygen saturation associations were more clearly expressed at 60 minutes, suggesting potential delayed responsiveness to higher-intensity light peaks.

Respiratory rate demonstrated consistent light-related increases (+1.11 breaths/min for visible light; +1.02 for peak light), with smaller contributions from temperature and noise. Oxygen saturation showed modest positive contrasts for light exposure (~+0.8%) and minimal temperature influence. Environmental modulation of respiratory variables was moderate in magnitude and broadly consistent across light metrics (Table S7).

***Core Body Temperature.***

After quality control and 15-minute aggregation, 731 rectal temperature observations were analyzed. Model performance was excellent (adjusted R² = 0.910) (Table S5).

Visible light exposure demonstrated a significant delayed association with rectal temperature, particularly at 30 minutes, with borderline early effects at 15 minutes. Ambient temperature and noise did not show independent associations after age and temporal adjustment.

The cyclic circadian component remained significant, indicating preserved residual 24-hour rhythmicity despite critical illness and environmental modulation. No significant linear temporal trend across PICU stay was observed for rectal temperature.

Sensitivity analysis using peak light exposure confirmed a delayed association pattern, with significant effects at 60 minutes and preserved circadian rhythmicity (Table S6). Overall, absolute temperature changes across environmental exposure ranges were modest, consistent with buffered thermoregulatory control under intensive care conditions.

Rectal temperature exhibited small percentile contrasts overall. Visible light was associated with modest decreases (−0.40 °C for visible light; −0.52 °C for peak light), whereas temperature and noise effects were minimal. Compared with cardiovascular and respiratory outcomes, thermoregulatory responses appeared relatively buffered (Table S7).

**Table S6. Summary of age-adjusted generalized additive models (GAMMs) including distributed lags of visible light and PICU Day.**

| **Outcome** | **n** | **Adjusted R²** | **Deviance explained** | **Temperature** | **Noise** | **Visible light distributed lags 15–60 min)** | **Circadian term (HOD)** | **PICU Day (β/day)** |
| --- | --- | --- | --- | --- | --- | --- | --- | --- |
| Heart rate (bpm) | 2248 | 0.781 | 78.5% | ✓ | ✓ | ✓ at 15 min; | Yes | +3.02 bpm/day |
| Systolic BP (mmHg) | 2006 | 0.563 | 57.0% | ✓ | NS | ✓ at 60 min | NS | +1.76 mmHg/day |
| Diastolic BP (mmHg) | 2004 | 0.292 | 30.2% | ✓ | NS | ✓ at 60 min ;; | Yes | +1.71 mmHg/day |
| Mean arterial pressure (mmHg) | 2130 | 0.329 | 33.9% | ✓ | NS | ✓ at 15 min and 60 min | NS | +1.12 mmHg/day |
| Respiratory rate (breaths/min) | 2118 | 0.599 | 60.7% | ✓ | ✓ | ✓ at 15 min | Yes | +0.67 breaths/min/day |
| Oxygen saturation (%) | 2055 | 0.841 | 84.4% | ✓ | ✓ | ✓ at 15 min | Yes | +0.45%/day |
| Rectal temperature (ºC) | 731 | 0.910 | 91.4% | NS | NS | ✓ at 30 min; | Yes | NS |

***Table S6. Summary of age-adjusted generalized additive models (GAMMs) including distributed lags of visible light and PICU Day.*** *Adjusted R² represents the proportion of outcome variability explained by each model, and “Deviance explained” reflects the percentage of total deviance accounted for by the fitted model. All models included age as a continuous covariate (years), PICU Day as a linear term (when applicable), a cyclic cubic spline for hour of day (HOD; 0–24 h) to model residual circadian rhythmicity, and a patient-level random intercept to account for repeated measures. Environmental predictors (ambient temperature, noise, and visible light intensity) were modeled using penalized cubic regression splines. Visible light exposure was specified using distributed lag smooth terms at 15, 30, 45, and 60 minutes. ✓ indicates a statistically significant non-linear association (p < 0.05); “Borderline” indicates marginal statistical significance (0.05 ≤ p < 0.10); NS indicates non-significant association. “PICU Day (β/day)” represents the estimated linear change in the outcome per additional day of PICU stay. Estimates are expressed in the natural units of each physiological variable.*

**Table S7. Summary of age-adjusted generalized additive mixed models (GAMMs) including distributed lags of peak light exposure and PICU Day.**

| **Outcome** | **n** | **Adjusted R²** | **Deviance explained** | **Temperature** | **Noise** | **Peak light (distributed lags 15–60 min)** | **Circadian term (HOD)** | **PICU Day (β/day)** |
| --- | --- | --- | --- | --- | --- | --- | --- | --- |
| **Heart rate (bpm)** | 2248 | 0.774 | 77.8% | ✓ | ✓ | ✓ at 15 and 30 min | Yes | +3.01 bpm/day |
| **Systolic BP (mmHg)** | 2006 | 0.561 | 56.8% | ✓ | Borderline | NS | NS | +1.75 mmHg/day |
| **Diastolic BP (mmHg)** | 2004 | 0.290 | 30.1% | ✓ | NS | Borderline at 60 min | Borderline | +1.71 mmHg/day |
| **Mean arterial pressure (mmHg)** | 2130 | 0.322 | 33.2% | ✓ | NS | NS | NS | +1.08 mmHg/day |
| **Respiratory rate (breaths/min)** | 2118 | 0.598 | 60.7% | ✓ | ✓ | ✓ at 15 and 45 min | Yes | +0.656 breaths/min/day |
| **Oxygen saturation (%)** | 2055 | 0.841 | 84.4% | ✓ | ✓ | ✓ at 60 min | Yes | +0.463 %/day |
| **Rectal temperature (ºC)** | 731 | 0.911 | 91.5% | NS | NS | ✓ at 60 min | Yes | NS |

***Table S7. Summary of age-adjusted generalized additive mixed models (GAMMs) including distributed lags of peak light exposure and PICU Day.*** *Adjusted R² represents the proportion of outcome variability explained by each model, and “Deviance explained” reflects the percentage of total deviance accounted for by the fitted model. All models included age as a continuous covariate (years), PICU Day as a linear term (when applicable), a cyclic cubic spline for hour of day (HOD; 0–24 h) to model residual circadian rhythmicity, and a patient-level random intercept to account for repeated measures. Environmental predictors (ambient temperature, noise, and peak light intensity were modeled using penalized cubic regression splines. Peak light exposure was specified using distributed lag smooth terms at 15, 30, 45, and 60 minutes. ✓ indicates a statistically significant non-linear association (p < 0.05); “Borderline” indicates marginal statistical significance (0.05 ≤ p < 0.10); NS indicates non-significant association. “PICU Day (β/day)” represents the estimated linear change in the outcome per additional day of PICU stay. Estimates are expressed in the natural units of each physiological variable.*

**Table S8. Percentile-based contrasts (ΔP75–P25) from age-adjusted GAMMs comparing visible light and peak light exposure**

| **Outcome** | **Temperature Δ** | **Visible light Δ** | **Visible light Δ (Peak)** | **Noise Δ** |
| --- | --- | --- | --- | --- |
| **Heart rate** | +4.81 | −6.37 | −5.14 | +1.43 |
| **Systolic BP** | −1.67 | +0.41 | +0.06 | −2.31 |
| **Diastolic BP** | −2.11 | −0.56 | −0.54 | −1.54 |
| **Mean arterial pressure** | −1.32 | −0.91 | −1.23 | −0.89 |
| **Respiratory rate** | −0.16 | +1.11 | +1.02 | −1.34 |
| **Oxygen saturation** | +0.08 | +0.79 | +0.82 | −0.73 |
| **Rectal temperature (°C)** | −0.03 | −0.40 | −0.52 | +0.61 |

***Table S8. Percentile-based contrasts (ΔP75–P25) from age-adjusted GAMMs comparing visible light and peak light exposure.*** *ΔP75–P25 values represent the expected change in outcome between the 75th and 25th percentiles of each environmental exposure. Light contrasts correspond to joint distributed-lag shifts (15–60 min). All models were age-adjusted and included PICU Day, cyclic HOD, patient random intercept, and AR(1) correction.*

**S6. Age-Stratified Generalized Additive Models (<2 vs ≥2 years).**

To explore potential effect modification by developmental stage, generalized additive mixed models were re-estimated separately for children aged <2 years and ≥2 years. Model specification was identical to whole-cohort analyses and included smooth terms for environmental exposures (ambient temperature, noise, and visible light using distributed lags at 15–60 min), a cyclic spline for hour of day (HOD), PICU Day as a linear term (when included), and a patient-level random intercept.

Model summaries for each age group are presented in Tables S8A–S8B

***Cardiovascular outcomes.***

In children ≥2 years (n ranging from 785 to 1,030 observations depending on outcome), model fit was highest for heart rate (adjusted R² = 0.881; deviance explained = 88.6%) and moderate for systolic blood pressure (adjusted R² = 0.586), whereas diastolic blood pressure and mean arterial pressure showed lower explanatory performance (adjusted R² = 0.294 and 0.287, respectively) (Table S8A). Ambient temperature was significantly associated with all cardiovascular outcomes in this group. Noise effects differed by outcome, showing significant associations for heart rate, diastolic blood pressure, and mean arterial pressure, but not for systolic blood pressure. Visible light demonstrated lag-specific associations across cardiovascular outcomes, with significant effects at 15 min for heart rate and at 30 min for systolic blood pressure; diastolic blood pressure showed significant effects at 60 min, while mean arterial pressure showed a borderline association at 30 min. Circadian modulation (HOD) was preserved for heart rate, systolic blood pressure, and mean arterial pressure, but not for diastolic blood pressure. PICU Day was associated with positive temporal trends for heart rate (+6.91 bpm/day), systolic blood pressure (+1.53 mmHg/day), and diastolic blood pressure (+2.01 mmHg/day), whereas the PICU Day slope for mean arterial pressure was small and non-significant (+0.35 mmHg/day, NS) (Table S8A).

In children <2 years (n ranging from 1,218 to 1,229 observations), model fit was moderate for heart rate (adjusted R² = 0.683) and systolic blood pressure (0.576), lower for diastolic blood pressure (0.284), and comparatively higher for mean arterial pressure (0.388) (Table S8B). Ambient temperature was significantly associated with heart rate but was non-significant for systolic blood pressure, mean arterial pressure, and rectal temperature, and borderline for diastolic blood pressure. Noise showed significant associations for heart rate and both systolic and diastolic blood pressure and was borderline for mean arterial pressure. Visible light exhibited consistent lagged associations for arterial pressure variables in this younger group, with significant effects at 15 and 60 minutes for systolic blood pressure, diastolic blood pressure, and mean arterial pressure; heart rate also showed a significant association at 15 minutes with borderline effects at 45 minutes. Circadian modulation (HOD) was preserved for heart rate and systolic blood pressure and was borderline for diastolic blood pressure, whereas mean arterial pressure did not show an independently detectable circadian component in this group (Table S8B). PICU Day was associated with a decrease in heart rate (−1.48 bpm/day) and positive temporal trends for arterial pressure variables (+2.46 mmHg/day for systolic BP, +1.58 mmHg/day for diastolic BP, and +2.14 mmHg/day for mean arterial pressure).

In children ≥2 years, cardiovascular responses showed larger environmental contrasts, particularly for heart rate, which exhibited marked modulation by noise (+16.00 bpm) and visible light (−8.29 bpm for visible light; −6.19 bpm for peak light), with temperature also contributing (+5.76 bpm) (Table S9A). Arterial pressure variables showed more modest but directionally consistent decreases with increasing noise and temperature. In children <2 years, cardiovascular contrasts were generally attenuated. Heart rate remained responsive to temperature (+4.46 bpm) and noise (+3.30 bpm), but light-related effects were smaller. Blood pressure responses were modest in magnitude, with slightly greater sensitivity to temperature (notably diastolic BP −3.12 mmHg) than to light (Table S9B).

***Respiratory variables.***

In children ≥2 years, respiratory rate demonstrated significant non-linear associations with ambient temperature and noise, as well as significant lagged associations with visible light at 15 minutes (Table S8A). Model performance was strong for respiratory rate (adjusted R² = 0.721; deviance explained = 73.1%), and PICU Day showed a positive temporal trend (+0.96 breaths/min/day). Oxygen saturation showed lower model performance (adjusted R² = 0.352) and was not independently associated with temperature or noise, but retained a significant association with visible light at 15 minutes; neither circadian modulation (HOD) nor the noise/temperature smooths were significant for SpO₂ in this age group. PICU Day showed a significant positive trend for oxygen saturation (+0.52%/day) (Table S8A).

In children <2 years, respiratory rate was significantly associated with temperature and noise, but did not show significant associations with visible light at any lag (Table S8B). Model fit for respiratory rate was modest (adjusted R² = 0.383), and the PICU Day slope was positive but small (+0.33 breaths/min/day). In contrast, oxygen saturation exhibited excellent model performance in this group (adjusted R² = 0.874; deviance explained = 87.8%) and showed significant associations with all environmental exposures (temperature, noise, and visible light), with visible light effects at 15 and 60 minutes. Circadian modulation (HOD) was preserved for both respiratory outcomes, and PICU Day showed a significant positive temporal trend for oxygen saturation (+0.45%/day) (Table S8B).

In older children (≥2 years), respiratory rate increased with temperature (+1.94 breaths/min) and noise (+12.52 breaths/min), while visible light was associated with small decreases. Oxygen saturation showed minimal environmental modulation. In contrast, in children <2 years, respiratory rate decreased with temperature (−1.84 breaths/min) and noise (−1.29 breaths/min) but increased with light exposure (≈+2.5 breaths/min for both visible light and peak light). Oxygen saturation in younger children demonstrated greater light sensitivity (+1.19% visible light; +1.61% peak light) compared with older children (Table S9A-S9B).

***Core body temperature.***

In children ≥2 years, rectal temperature showed excellent model fit (adjusted R² = 0.973; deviance explained = 97.7%) and was significantly associated with ambient temperature and visible light (15 min), with noise showing borderline significance (Table S8A). Circadian modulation (HOD) remained clearly detectable, and a small but statistically significant positive PICU Day trend was observed (+0.08 °C/day).

In children <2 years, rectal temperature showed substantially lower model performance (adjusted R² = 0.229; deviance explained = 26.1%) and did not show independent associations with temperature, noise, or visible light in the stratified models (Table S8B). Nevertheless, circadian rhythmicity (HOD) remained detectable, and no PICU Day trend was observed.

In children ≥2 years, rectal temperature showed small decreases with ambient temperature (−0.39 °C) and small increases with visible light (+0.44 °C visible light; +0.27 °C peak light), while noise had negligible impact. In children <2 years, temperature responses differed, showing a positive contrast with ambient temperature (+0.77 °C) and minimal modulation by light or noise. Overall, thermoregulatory responses were modest in magnitude in both age groups. Detailed percentile-based are shown in Tables S9A-S9B.

**Table S9A. Age-stratified generalized additive mixed models (GAMMs) in children aged ≥2 years**

| **Outcome** | **n** | **Adjusted R²** | **Deviance explained** | **Temperature** | **Noise** | **Visible Light(lags 15–60 min)** | **Circadian term (HOD)** | **PICU Day (β/day)** |
| --- | --- | --- | --- | --- | --- | --- | --- | --- |
| Heart rate (bpm) | 1030 | 0.881 | 88.6% | ✓ | ✓ | ✓ at 15 min | Yes | +6.91 bpm/day |
| Systolic BP (mmHg) | 786 | 0.586 | 60.0% | ✓ | NS | ✓ at 30 min; | Yes | +1.53 mmHg/day |
| Diastolic BP (mmHg) | 785 | 0.294 | 31.7% | ✓ | ✓ | ✓ at 60 min; | NS | +2.01 mmHg/day |
| Mean arterial pressure (mmHg) | 901 | 0.287 | 30.9% | ✓ | ✓ | Borderline at 30 min | Yes | +0.35 mmHg/day (NS) |
| Respiratory rate (breaths/min) | 1006 | 0.721 | 73.1% | ✓ | ✓ | ✓ at 15 min | Yes | +0.96 breaths/min/day |
| Oxygen saturation (%) | 855 | 0.352 | 36.5% | NS | NS | ✓ at 15 min | NS | +0.52%/ day |
| Rectal temperature (ºC) | 305 | 0.973 | 97.7% | ✓ | Borderline | ✓ at 15 min | Yes | +0.08 ºC/day |

**Table S9B. Age-stratified generalized additive mixed models (GAMMs) in children aged <2 years**

| **Outcome** | **n** | **Adjusted R²** | **Deviance explained** | **Temperature** | **Noise** | **Visible light (lags 15–60 min)** | **Circadian term (HOD)** | **PICU Day (β/day)** |
| --- | --- | --- | --- | --- | --- | --- | --- | --- |
| Heart rate (bpm) | 1218 | 0.683 | 69.1% | ✓ | ✓ | ✓ at 15 min; 45 borderline | Yes | −1.48 bpm/day |
| Systolic BP (mmHg) | 1220 | 0.576 | 58.3% | NS | ✓ | ✓ at 15 and 60 min | Yes | +2.46 mmHg/day |
| Diastolic BP (mmHg) | 1219 | 0.284 | 29.9% | Borderline | ✓ | ✓ at 15 and 60 min | Borderline | +1.58 mmHg/day |
| Mean arterial pressure (mmHg) | 1229 | 0.388 | 39.7% | NS | Borderline | ✓ at 15 and 60 min | NS | +2.14 mmHg/day |
| Respiratory rate (breaths/min) | 1112 | 0.383 | 40.0% | ✓ | ✓ | NS | Yes | +0.33 breaths/min/day |
| Oxygen saturation (%) | 1200 | 0.874 | 87.8% | ✓ | ✓ | ✓ at 15 min and 60 min | Yes | +0.45%/day |
| Rectal temperature (ºC) | 426 | 0.229 | 26.1% | NS | NS | NS | Yes | NS |

***Table S9A-S9B. Age-stratified generalized additive mixed models (GAMMs) in children aged ≥2 years (S8A) and <2 years (S8B).*** *Adjusted R² represents the proportion of outcome variability explained by each model, and “Deviance explained” reflects the percentage of total deviance accounted for by the fitted model. All models included smooth terms for ambient temperature (AMBT), noise (dB), and visible light intensity, specified as distributed lag smooths at 15, 30, 45, and 60 minutes. A cyclic cubic spline for hour of day (HOD; 0–24 h) was used to model residual circadian rhythmicity, and a patient-level random intercept accounted for repeated measurements. PICU Day was included as a linear term when non-constant.*

*✓ indicates a statistically significant non-linear association (p < 0.05); “Borderline” indicates marginal statistical significance (0.05 ≤ p < 0.10); NS indicates non-significant association. “PICU Day (β/day)” represents the estimated linear change in the outcome per additional PICU day. Estimates are expressed in the natural units of each physiological variable.*

**Table S10A. Percentile-based contrasts (ΔP75–P25) from age-stratified (≥2 years) age-adjusted GAMMs comparing visible light and peak light exposure**

| **Outcome** | **Temperature Δ** | **Visible light Δ** | **Visible light Δ (Peak)** | **Noise Δ** |
| --- | --- | --- | --- | --- |
| Heart rate (bpm) | +5.76 | −8.29 | −6.19 | +16.00 |
| Systolic BP (mmHg) | −3.37 | −0.67 | −0.25 | −5.25 |
| Diastolic BP (mmHg) | −1.05 | −1.47 | −0.44 | −9.02 |
| Mean arterial pressure (mmHg) | −1.84 | −0.92 | −0.09 | −4.83 |
| Respiratory rate (breaths/min) | +1.94 | −1.32 | −1.46 | +12.52 |
| Oxygen saturation (%) | +0.09 | +0.31 | +0.37 | −0.69 |
| Rectal temperature (°C) | −0.39 | +0.44 | +0.27 | +0.02 |

**Table S10B. Percentile-based contrasts (ΔP75–P25) from age-stratified (<2 years) age-adjusted GAMMs comparing visible light and peak light exposure**

| **Outcome** | **Temperature Δ** | **Visible light Δ** | **Visible light Δ (Peak)** | **Noise Δ** |
| --- | --- | --- | --- | --- |
| Heart rate | +4.46 | −3.09 | −1.28 | +3.30 |
| Systolic BP (mmHg) | −0.54 | +0.33 | +0.93 | −2.72 |
| Diastolic BP (mmHg) | −3.12 | −0.53 | −0.84 | −2.43 |
| Mean arterial pressure (mmHg) | −1.88 | −0.81 | −0.93 | −2.17 |
| Respiratory rate (breaths/min) | −1.84 | +2.56 | +2.41 | −1.29 |
| Oxygen saturation (%) | +0.29 | +1.19 | +1.61 | −0.54 |
| Rectal temperature (°C) | +0.77 | −0.14 | −0.13 | +0.07 |

***Table S10A–S10B. Percentile-based contrasts (ΔP75–P25) from age-stratified age-adjusted GAMMs (≥2 years and <2 years).*** *ΔP75–P25 values represent the estimated change in each outcome when the exposure increases from the 25th to the 75th percentile. Models were stratified by age group (S7A: ≥2 years; S7B: <2 years) and included PICU Day (linear term, when applicable), a cyclic spline for hour of day (HOD), a patient-level random intercept, and AR(1) correction (ρ = 0.3). Temperature and noise were modeled using penalized splines. Visible light and peak light contrasts correspond to joint distributed-lag shifts (15–60 min). Estimates are expressed in the natural units of each outcome*
